# Study on the Dynamic Characteristics of a SiC-Based Capacitive Micro-Accelerometer in Rarefied Air

**DOI:** 10.3390/ma15134692

**Published:** 2022-07-04

**Authors:** Xiang Tian, Wei Sheng

**Affiliations:** School of Instrumentation and Optoelectronic Engineering, Beihang University, Beijing 100191, China

**Keywords:** micro-accelerometer, rarefied air, squeeze-film damping, dynamic characteristics

## Abstract

In this study, we investigated the viscosity, squeeze-film damping, and a SiC-based capacitive micro-accelerometer in rarefied air. A specific expression for the effective viscosity coefficient of the air was derived, and when the air pressure drops from the standard atmospheric pressure, the viscosity of the air will decrease accordingly. Decreases in the air pressure and the viscosity of the air lead to the change in the squeeze-film air damping in the micro-accelerometer, and both the viscous damping force and the elastic damping force of the air film between the moving electrode plate and the fixed electrode plate will also decrease. The damping coefficient and relative damping ratio of the micro-accelerometer in rarefied air were calculated, which was also confirmed by simulations. The changes of the damping coefficient and the relative damping ratio of the system will directly affect the dynamic characteristics of the micro-accelerometer. When the air pressure in the working environment is below the standard atmospheric pressure, the micro-accelerometer will be in an underdamping state. With the decrease in the air pressure, the working bandwidth of the micro-accelerometer will decrease significantly, and the resonant phenomenon may appear. However, the decrease in the air pressure will not have a notable impact on the response time of the micro-accelerometer. Therefore, this work provides a theoretical basis for the study of the performance characteristics of a SiC-based capacitive accelerometer in rarefied air.

## 1. Introduction

Since about 1946, due to the possibility of high-speed flight in the high-altitude field, researchers have greatly increased their interest in rarefied gas dynamics [1]. The newly developed aircraft spend more and more time flying in rarefied gas, and it is important to understand the performance characteristics of various electronic devices of aircraft flying in rarefied gas [2,3]. In MEMS sensors, the microstructures usually have the characteristics of some small gaps between the moving elements and the substrates [4,5]. When the gas pressure is low, the mean free path of gas molecules and the gap distance are comparable, so the viscosity coefficient of gas will be changed accordingly [6,7]. The models of the rarefied gas flow in the small gap have been developed since the middle of the last century [8,9,10,11].

In the 1960s, Cercignani et al. from the University of Milano introduced a general variational principle applied to kinetic models and utilized it to solve the Poiseuille flow problem of a rarefied gas between two parallel plates [12,13]. In the 1980s, a generalized Reynolds equation in the lubrication theory was derived from a linearized Boltzmann equation by Fukui and Kaneko, and the effective viscosity of gas at low pressures was quantified by an approximate expression [14,15,16]. In 1995, Veijola et al. of Helsinki University of Technology proposed a novel electric equivalent circuit for the air damping forces created by the squeeze-film damping between the parallel plates. The circuit model was also used to calculate the effective viscosity in the gap between the parallel plates [17].

In 2009, Mol et al. performed an experiment on the squeeze-film effect in rarefied air and compared the experimental results with the molecular dynamics model and the model based on the modified Reynolds equation [18]. In 2010, a 3D simulation approach was proposed by Leung et al. for modeling the squeeze-film damping on flexible micro-resonators in highly rarefied air. Compared with the existing methods, this simulation method was more general, and it could simulate resonators of various shapes [19]. In 2020, an analytical model was established for the squeeze-film effect on the perforated plate structure, which had been widely used in MEMS sensors. Compared with the existing damping models, the model could be applicable to a wider geometry range [20].

**Figure 1:**
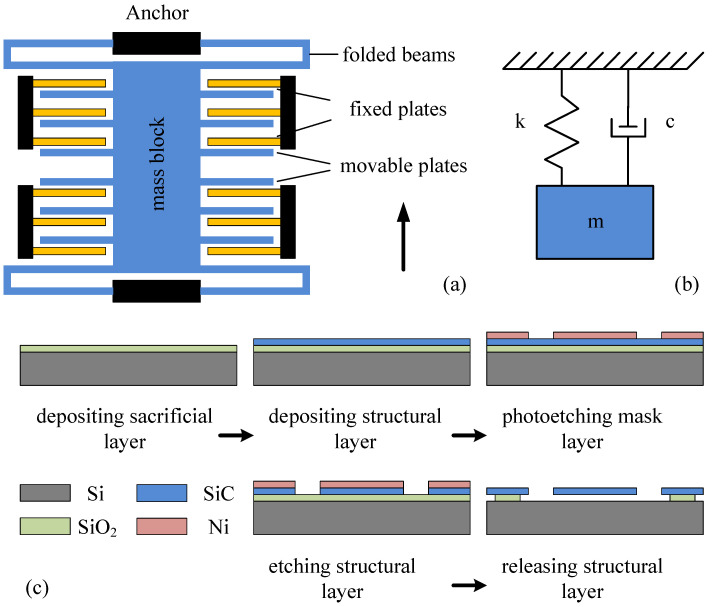
Model of a single-axis, comb-type capacitive micro-accelerometer. (**a**) Physical model of the accelerometer, (**b**) mechanical model of the accelerometer, and (**c**) process flow of the accelerometer [21].

In [21], we designed a capacitive micro-accelerometer based on a silicon carbide microstructure by the finite element method (FEM). The physical model and the process flow of the accelerometer are shown in Figure 1, and the optimized structural parameters are listed in Table 1. Silicon carbide has the advantages of excellent mechanical durability and thermal stability, so it was deposited as the structural layer of micro-accelerometer by the low-pressure chemical vapor deposition (LPCVD) technique. In this case, the accelerometer had the characteristics of high natural frequency, wide range, favorable dynamic characteristics, and remarkable high-temperature resistance. When the sensitive structure of the micro-accelerometer moves in the air, squeeze-film air damping will be produced between the fixed electrode plate and the moving electrode plate, which may affect the dynamic performances of the micro-accelerometer in a low-pressure environment. Therefore, it is necessary for this impact to make an accurate theoretical analysis and a necessary simulation verification, in order to obtain the conclusion of long-term use of the micro-accelerometer in a low-pressure environment.

In this paper, we study the viscosity, squeeze-film effect, and a SiC-based capacitive micro-accelerometer in rarefied air. When the air pressure drops from the standard atmospheric pressure, the viscosity of the air will decrease accordingly. The decreases in the air pressure and the viscosity of the air lead to the decrease in the damping coefficient and relative damping ratio of the micro-accelerometer, and the change in the dynamic characteristics of the micro-accelerometer. With the decrease in the air pressure, the working bandwidth of the micro-accelerometer will decrease significantly, but it will not have a great impact on the response time of the micro-accelerometer.

## 2. Effective Viscosity in the Rarefied Air

### 2.1. Effective Viscosity Coefficient

At the standard atmospheric pressure, the air can be regarded as a viscous fluid due to frequent collisions between air molecules. The experimental results show that the effect of air damping is quite constant when the air pressure is close to the standard atmospheric pressure. Nevertheless, when the air is rarefied to a pressure well below the atmospheric pressure, the air damping decreases significantly.

At present, two basic methods have been used to consider the damping in rarefied air: the “effective viscosity coefficient” and the free molecule model [22,23,24]. The first method proposed that the governing equation of squeeze-film air damping is still valid in rarefied air, but the “viscosity coefficient” should be replaced by an effective coefficient ($\mu_{eff}$), which depends on the air pressure through the Knudsen number ($K_{n}$) of the system. The Knudsen number is an important parameter for the fluid in micro- and nano-scale structures; it is defined as the ratio of the mean free path of fluid molecules to the typical size of the structure [25].

(1) $$K_{n}=\frac{\lambda }{h}$$

Here, λ represents the mean free path of the fluid molecules. For the air, the mean free path of molecules refers to the average distance that molecules travel between two collisions. h is the typical size of the damping structure. For the squeeze-film air damping, it is the distance between the plate and the substrate.

The mean free path of air molecules can be written as [26]

(2) $$\lambda =\frac{1}{\sqrt{2}\pi nd^{2}}$$

where n is the number density of air molecules and d is the diameter of the air molecules.

According to the relationship between the air pressure and the temperature,

(3) $$p=nk_{B}T$$

where $k_{B}$ is the Boltzmann constant, and the mean free path of air molecules can be written as follows:

(4) $$\lambda =\frac{k_{B}T}{\sqrt{2}\pi d^{2}p}$$

By substituting Equation (4) into Equation (1), the Knudsen number of the air in the MEMS structure can be obtained:

(5) $$K_{n}=\frac{k_{B}T}{\sqrt{2}\pi d^{2}ph}$$

According to the work of Veijola et al. [17], an approximate equation for the effective viscosity coefficient could be obtained:

(6) $$\mu_{eff}=\frac{\mu_{0}}{1+9.638K_{n}^{1.159}}=\frac{\mu_{0}}{1+9.638\times {\left(\frac{k_{B}T}{\sqrt{2}\pi d^{2}ph}\right)}^{1.159}}$$

where $\mu_{0}$ is the viscosity coefficient at the standard atmospheric pressure.

### 2.2. Analysis of Effective Viscosity

According to the value of the Knudsen number, the nature of the air flow area can be judged. Generally speaking, air flow can be divided into four zones. When $K_{n}\leq 10^{-3}$, the air flow is in the continuous zone; when $10^{-3}<K_{n}\leq 0.1$, the air flow is in the slip zone; when $0.1<K_{n}\leq 10$, the air flow is in the transition zone; and when $K_{n}>10$, the air flow is in the free molecule zone. When the air flows in the continuous zone, it can be described by the governing equation based on the continuous medium assumption. If the viscous effect of air is not considered, the Euler equation is used. If the viscous effect of air is to be considered, the Navier–Stokes (N-S) equation and no-slip boundary condition are used. When the air flows in the slip zone, it gradually deviates from the thermodynamic equilibrium, but the air can still be described by the N-S equation, and the slip boundary conditions need to be adopted on the boundary. When the air flows in the free molecule zone, the particle dynamics methods must be used to study the air movement, such as the direct simulation Monte Carlo (DSMC) method. Finally, when air flows in the transition zone, it can be regarded neither as a pure continuous medium nor as a free molecule flow. At this time, the simulation calculation of air is difficult [25].

In the study, the rarefied air in the continuous zone and the slip zone are considered, and Equation (6) is used for the analysis of the effective viscosity. The Boltzmann constant is $k_{B}=1.38\times 10^{-23}\;J/K$, and the ambient temperature is at room temperature (T=15 °C). Generally speaking, the effective diameter of the gas molecule is related to the molecular type, which is basically in the range of $d=2\sim 6\times 10^{-10}\;m$.

**Figure 2:**
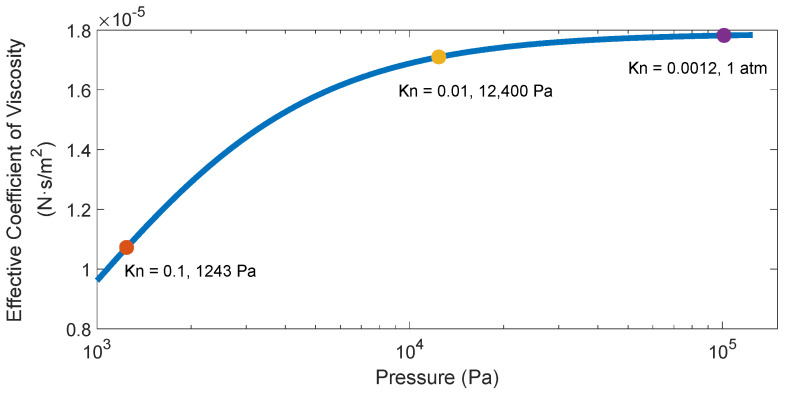
The dependence of the effective viscosity coefficient of air on the air pressure.

Figure 2 shows the relationship between the effective viscosity coefficient of the air and the air pressure when the air pressure is lower than the standard atmospheric pressure. The variation values of the relevant parameters of air can be seen in Table 2. When the air pressure is the standard atmospheric pressure, the mean free path of air molecules is $2.45\times 10^{-8}\;m$. At this time, the Knudsen number of air is $K_{n}=0.0012$, so the effective viscosity coefficient of air is $\mu_{eff}=1.78\times 10^{-5}\;N\cdot s/m^{2}$. When the air pressure drops to 12,400 Pa, the mean free path of air molecules becomes $2.01\times 10^{-7}\;m$. At this time, the Knudsen number of air is $K_{n}=0.01$, so the effective viscosity coefficient of air becomes $\mu_{eff}=1.71\times 10^{-5}\;N\cdot s/m^{2}$. When the air pressure drops to 1243 Pa, the mean free path of air molecules is $2\times 10^{-6}\;m$. At this time, the Knudsen number of air is $K_{n}=0.1$, so the effective viscosity coefficient of air becomes $\mu_{eff}=1.07\times 10^{-5}\;N\cdot s/m^{2}$. It can be seen from the above data that when the air pressure decreases by an order of magnitude from the standard atmospheric pressure, the mean free path of the air molecules increases by one order of magnitude, the Knudsen number of the air also increases by one order of magnitude, and the effective viscosity coefficient of the air decreases accordingly. The calculation results show that when the air pressure drops from the standard atmospheric pressure ($K_{n}=0.0012$) to 12,400 Pa ($K_{n}=0.01$), the effective viscosity coefficient of the air decreases by about 4.1%, and the decline is not obvious, but when the air pressure drops from the standard atmospheric pressure ($K_{n}=0.0012$) to 1243 Pa ($K_{n}=0.1$), the effective viscosity coefficient of the air decreases by about 39.9%, and the decline is very obvious.

## 3. Squeeze-Film Air Damping in the Rarefied Air

In Section 2, we detailed the effect of the drop in the air pressure on the viscosity coefficient of the air. In this section, we examine the effect of the drop in the air pressure on the squeeze-film air damping in the MEMS accelerometer.

### 3.1. Squeeze-Film Effect

**Figure 3:**
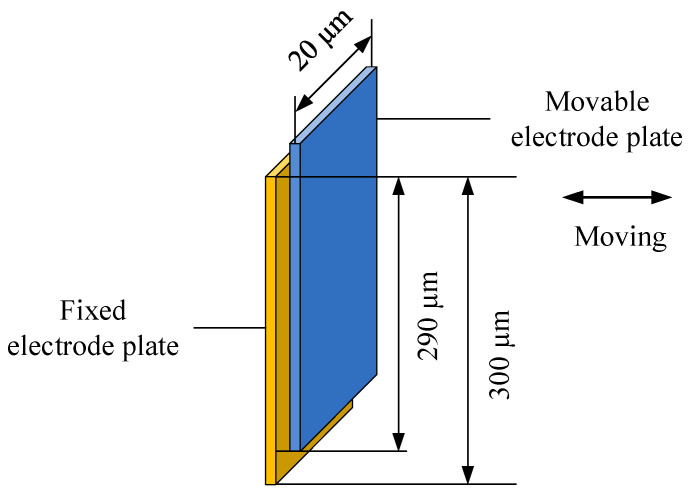
The comb structure in the micro-accelerometer.

As shown in Figure 3, when the comb-type capacitive accelerometer vibrates, squeeze-film air damping will be produced between the moving electrode plate and the fixed electrode plate. The length and the width of the overlapping area of the movable electrode plate and the fixed electrode plate are 290 µm and 20 µm, respectively, while the length and the width of both plates are 300 µm and 20 µm, respectively. The gap distance between the movable electrode plate and the fixed electrode plate is 1.23 µm. When the displacement of the movable plate is $h=h_{1}\cos(\omega t)$, the analytical expressions of the viscous damping force $F_{0}$ and elastic damping force $F_{1}$ of the rectangular plate can be derived [21]:

(7) $$F_{0}=\frac{64plw\sigma }{\pi^{6}}\frac{h_{1}}{h_{0}}\underset{m,n;odd}{\Sigma }\frac{m^{2}+{\left(n/\beta \right)}^{2}}{{\left(mn\right)}^{2}\{{\left[m^{2}+{\left(n/\beta \right)}^{2}\right]}^{2}+\sigma^{2}/\pi^{4}\}}$$

(8) $$F_{1}=\frac{64plw\sigma^{2}}{\pi^{8}}\frac{h_{1}}{h_{0}}\underset{m,n;odd}{\Sigma }\frac{1}{{\left(mn\right)}^{2}\{{\left[m^{2}+{\left(n/\beta \right)}^{2}\right]}^{2}+\sigma^{2}/\pi^{4}\}}$$

where l and w are the length and the width of the rectangular plate, respectively; β=w/l is the aspect ratio of the rectangular plate; $h_{0}$ is the gap distance between the movable electrode plate and the fixed electrode plate; p is the initial pressure in the comb structure; and $\sigma =12\mu \omega l^{2}/h_{0}^{2}p$ is the squeeze number.

**Figure 4:**
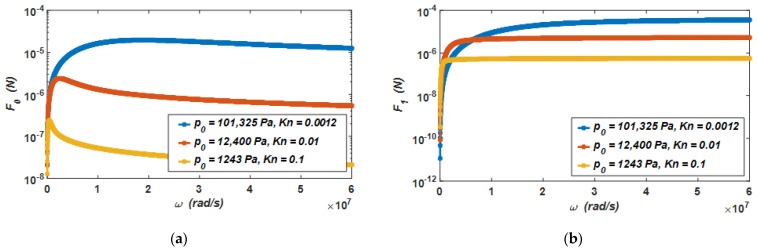
(**a**) The relationship between the viscous damping force and the vibration frequency of the micro-accelerometer, and (**b**) the relationship between the elastic damping force and the vibration frequency of the micro-accelerometer when the air pressure drops from the standard atmospheric pressure to 1243 Pa.

Additionally, it is assumed that the vibration amplitude of the movable electrode plate is $h_{1}=0.1\;\mu m$. According to Equations (7) and (8), when the air pressure drops from the standard atmospheric pressure ($K_{n}=0.0012$) to 1243 Pa ($K_{n}=0.1$), the relationship between the viscous damping force produced by the air film and the vibration frequency of the micro-accelerometer is shown in Figure 4a, and the relationship between the elastic damping force produced by the air film and the vibration frequency of the micro-accelerometer is shown in Figure 4b.

It can be seen from the curves in the two figures that when the air pressure drops from the standard atmospheric pressure ($K_{n}=0.0012$) to 1243 Pa ($K_{n}=0.1$), the viscous damping force and elastic damping force at different air pressures have the following characteristics. When the vibration frequency of the micro-accelerometer is low, the viscous damping force will increase linearly with the increase in the vibration frequency. At this time, the elastic damping force increases slowly. However, when the vibration frequency of the micro-accelerometer increases gradually, the viscous damping force decreases with the increase in the vibration frequency. At this time, the elastic damping force increases rapidly. The results provide evidence that when the micro-accelerometer vibrates, damping is the main form of the air film in the low-frequency vibration, and stiffness is the main form of the air film in the high-frequency vibration. In this capacitive micro-accelerometer, as it works at a low frequency, the viscous damping force is the main source of its air damping force.

Furthermore, the viscous damping force and elastic damping force of the air will decrease with the decrease in air pressure. When the air pressure decreases by one order of magnitude, the viscous damping force and elastic damping force of the air will also decrease by one order of magnitude.

### 3.2. Damping Coefficient and Relative Damping Ratio

The spring constant for the micro-accelerometer can be determined as [27]

(9) $$k=2ET\frac{W^{3}}{L^{3}}$$

where E is the Young’s modulus of the silicon carbide, and L, W, and T are the length, width, and thickness of the folded support beam, respectively.

The relative damping ratio is an important parameter of the micro-accelerometer, which can determine the dynamic characteristics of the system. Its expression is

(10) $$\varsigma =\frac{c}{2\sqrt{mk}}$$

where the damping coefficient c can be obtained by Equation (7) ($F_{0}=c\cdot v$), and the spring constant k can be derived from Equation (9).

**Figure 5:**
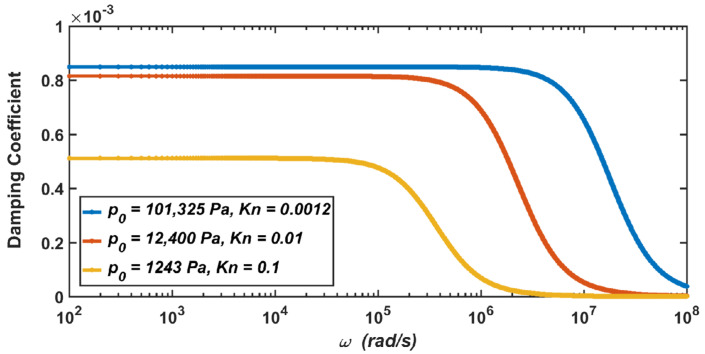
The dependence of the damping coefficient of the micro-accelerometer on the vibration frequency when the air pressure drops from the standard atmospheric pressure to 1243 Pa.

**Figure 6:**
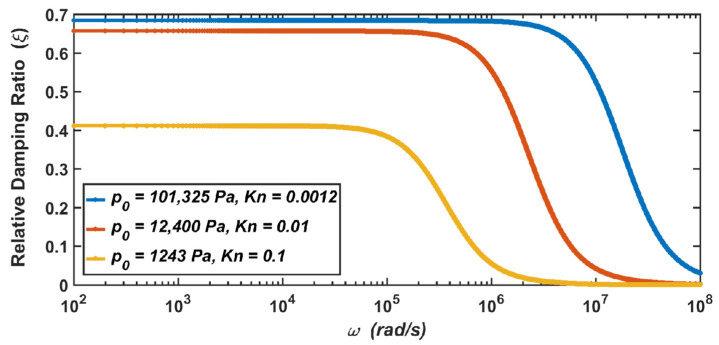
The dependence of the relative damping ratio of the micro-accelerometer on the vibration frequency when the air pressure drops from the standard atmospheric pressure to 1243 Pa.

Due to the small size and the light weight of the MEMS sensor, it is easily affected by the air, and it has a notable impact in most cases [28,29]. It can be seen from Equations (7) and (10) that when the air pressure drops, the damping coefficient and relative damping ratio of the system will change with the change in air pressure. After the calculation, when the air pressure drops from the standard atmospheric pressure ($K_{n}=0.0012$) to 1243 Pa ($K_{n}=0.1$), the relationship between the damping coefficient of the micro-accelerometer and the vibration frequency is shown in Figure 5, and the relationship between the relative damping ratio of the micro-accelerometer and the vibration frequency is shown in Figure 6.

The results show that when the micro-accelerometer is at the standard atmospheric pressure, its damping coefficient is $c=8.73\times 10^{-4}$, and its relative damping ratio is ς=0.7. When the micro-accelerometer works at 12,400 Pa, the damping coefficient and relative damping ratio become $c=8.16\times 10^{-4}$ and ς=0.66. When the micro-accelerometer works at 1243 Pa, the two parameters become $c=5.12\times 10^{-4}$ and ς=0.41. Therefore, it can be seen from the above results that with the decrease in the air pressure, the corresponding damping coefficient of the micro-accelerometer decreases, and the corresponding relative damping ratio also decreases.

It can be seen from Equation (7) that the damping coefficient is related to the air pressure and the viscosity coefficient of the air. As shown in Table 2 in Section 2.2, the decrease in the viscosity coefficient of the air at 12,400 Pa is not obvious, while the decrease at 1243 Pa is obvious. Therefore, compared with the damping coefficient at the standard atmospheric pressure, the decrease in the damping coefficient at 12,400 Pa is not obvious, while the decrease at 1243 Pa is obvious. Similarly, the analysis is also applicable to the relative damping ratio of the micro-accelerometer.

### 3.3. Simulation of Squeeze-Film Air Damping

**Figure 7:**
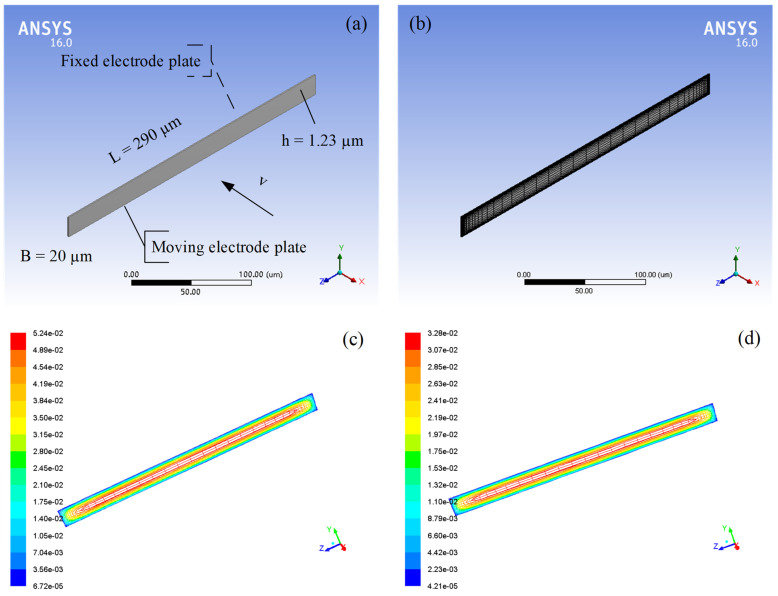
(**a**) Physical model and (**b**) meshing model of squeeze-film air damping between the fixed electrode plate and the moving electrode plate in the micro-accelerometer; the air pressure distributions between the fixed electrode plate and the moving electrode plate when the air pressure is (**c**) 12,400 Pa and (**d**) 1243 Pa.

In order to verify the theoretical analysis of the squeeze-film damping in the rarefied air, a physics-level simulation is proposed by Ansys/Fluent to simulate the squeeze-film air damping between the fixed electrode plate and moving electrode plate in the micro-accelerometer (the comb structure). As described in Section 3.1, it is assumed that the lengths and widths of the two plates are 290 µm and 20 µm, respectively, and the gap distance between them is 1.23 µm. The physical model of the comb structure is shown in Figure 7a, and the meshing model is shown in Figure 7b. When the moving electrode plate moves to the fixed electrode plate at a certain speed, the air between two plates is squeezed, and squeeze-film air damping will be produced.

After modeling and meshing the comb structure, some simulation parameters are set up, as listed in Table 3. When the air pressure is below the standard atmospheric pressure, the density and viscosity coefficient of the air will change with the change in air pressure. According to the ideal gas law (pV=nRT), the air density is directly proportional to the air pressure, so the air density at 12,400 Pa is 0.15 kg/m^3^, and the air density at 1243 Pa is 0.015 kg/m^3^. The viscosity coefficients of the air at different air pressures have been calculated, as shown in Section 2.2. The environment temperature is constant (15 °C). Moreover, it is assumed that the speed of the moving electrode plate is 10 µm/s, which can be set up through a profile file in Fluent.

Two groups of simulations under low pressures are carried out. One is when the air pressure is 12,400 Pa; the moving electrode plate moves towards the fixed electrode plate along the x-axis at the speed of 10 µm/s, and the air pressure distribution between the plates is shown in Figure 7c. The other is when the air pressure is 1243 Pa; the moving electrode plate moves towards the fixed electrode plate along the x-axis at the speed of 10 µm/s, and the air pressure distribution between the plates is shown in Figure 7d. It can be seen from both figures that high pressures appear in the middle of the air film and low pressures appear at the edges of the air film. There are two important parameters for the comb structure, including the air damping force of the moving electrode plate and the damping coefficient of the comb structure. The theoretical values of these two parameters can be obtained from Equation (7), and the simulated values can be calculated by Fluent, as shown in Table 4. There are 40 pairs of the comb structures in the micro-accelerometer, and the corresponding air damping force and the damping coefficient of the micro-accelerometer under low pressures are also shown in Table 4. These results show that the simulated values of the relevant parameters for the comb structure and the micro-accelerometer are close to the theoretical values, which effectively verifies the theoretical analysis of the squeeze-film air damping in rarefied air.

Based on the above analysis, the damping coefficient and the relative damping ratio of the micro-accelerometer decrease with the decrease in the air pressure, and the change will directly lead to the change in the dynamic characteristics of the micro-accelerometer, which includes amplitude–frequency characteristics and step responses. Therefore, we assume that the air pressure drops from the standard atmospheric pressure ($K_{n}=0.0012$) to 1243 Pa ($K_{n}=0.1$), and the micro-accelerometer in this condition is analyzed in the frequency domain and the time domain.

## 4. Dynamic Characteristic Analysis

### 4.1. Amplitude–Frequency Characteristics

According to the content of automatic control principle, the logarithmic amplitude–frequency characteristic of the MEMS sensor is

(11) $$L(\omega )=-20\mathrm{lg}\sqrt{{\left(1-\omega^{2}/\omega_{n}^{2}\right)}^{2}+4\varsigma^{2}\omega^{2}/\omega_{n}^{2}}$$

where $\omega_{n}=\sqrt{k/m}$ is the resonant frequency of the MEMS sensor, ω is the vibration frequency of the MEMS sensor, and ς is the relative damping ratio of the MEMS sensor.

**Figure 8:**
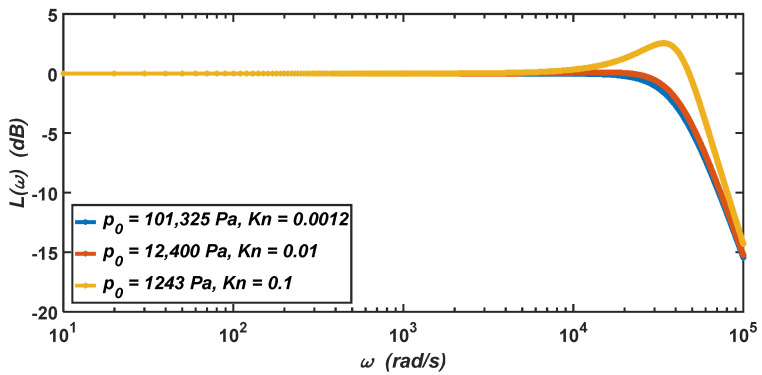
The logarithmic amplitude–frequency characteristics of the micro-accelerometer when the air pressure drops from the standard atmospheric pressure to 1243 Pa.

The amplitude–frequency characteristics of the micro-accelerometer when the air pressure decreases from the standard atmospheric pressure ($K_{n}=0.0012$) to 1243 Pa ($K_{n}=0.1$) are shown in Figure 8, which are only determined by the relative damping ratio of the micro-accelerometer. It can be seen from the figure that when the micro-accelerometer is at the standard atmospheric pressure, its relative damping ratio is ς=0.7, which will give the micro-accelerometer two advantages at the same time. The first advantage is that the micro-accelerometer has a large working bandwidth of about 0–30,000 Hz. The second advantage is that the amplitude–frequency characteristic curve of the micro-accelerometer has no resonant peak and the resonant phenomenon will not occur. When the air pressure drops to 12,400 Pa, the damping ratio will become ς=0.66. At this time, the working bandwidth of the micro-accelerometer increases to about 0–33,500 Hz, and its amplitude–frequency characteristic curve has almost no resonant peak. When the air pressure drops to 1243 Pa, the damping ratio will become ς=0.41. At this time, the amplitude–frequency characteristic curve of the micro-accelerometer has a resonant peak, and the working bandwidth drops to about 0–17,500 Hz.

In summary, when the air pressure in the working environment is below the standard atmospheric pressure, the micro-accelerometer will be in the underdamping state. With the decrease in the air pressure in the working environment, the working bandwidth of the micro-accelerometer will decrease significantly, and the resonant peak will appear in the amplitude–frequency characteristic curve—that is, the vibration amplitude of the micro-accelerometer will become very large, and the micro-accelerometer may be damaged when the vibration frequency approaches its resonant frequency.

### 4.2. Step Responses

As shown in Figure 1b, the comb-type capacitive micro-accelerometer could be equivalent to a second-order damping system:

(12) $$m\cdot \ddot{x}(t)+c\cdot \dot{x}(t)+k\cdot x(t)=m\cdot a(t)$$

where m, c, and k are the mass, damping coefficient, and spring constant of the micro-accelerometer, respectively. x(t) is the displacement of the micro-accelerometer, a(t) is the external acceleration, and t is time.

**Figure 9:**
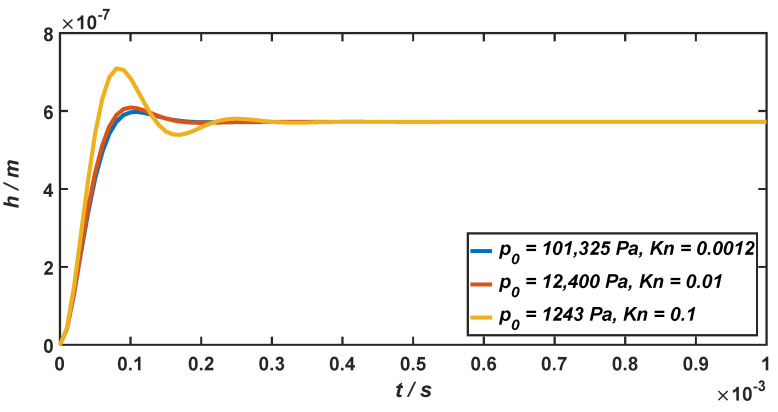
The step responses of the micro-accelerometer when the air pressure drops from the standard atmospheric pressure to 1243 Pa.

The step responses of the micro-accelerometer at the external acceleration of 100 g when the air pressure of the working environment drops from the standard atmospheric pressure ($K_{n}=0.0012$) to 1243 Pa ($K_{n}=0.1$) are shown in Figure 9, which are only determined by the relative damping ratio of the micro-accelerometer.

As illustrated in Figure 9, when the micro-accelerometer is at the standard atmospheric pressure, its relative damping ratio is ς=0.7, and the response time is $RT=4\times 10^{-4}\;s$. When the air pressure drops to 12,400 Pa and 1243 Pa, the relative damping ratio will become ς=0.66 and ς=0.41, and the response time will become $RT=5\times 10^{-4}\;s$ and $RT=7\times 10^{-4}\;s$. It can be seen from the above results that when the air pressure of the working environment is below the standard atmospheric pressure, the micro-accelerometer will be in the underdamping state. With the decrease in the air pressure in the working environment, the response time of the micro-accelerometer will increase accordingly. When the air pressure decreases from 101,325 Pa to 1243 Pa, the response time of the system increases to 1.75 times. However, since it is still in the same order of magnitude (10^−4^ s), the decrease in the air pressure will not have a considerable impact on the response time of the micro-accelerometer.

## 5. Discussion

In this work, we studied the viscosity in rarefied air and derived a specific expression for the effective viscosity coefficient of the air. When the air pressure decreases by an order of magnitude from the standard atmospheric pressure, the mean free path of the air molecules increases by one order of magnitude; the Knudsen number of air also increases by one order of magnitude, and the effective viscosity coefficient of the air decreases accordingly.

The decreases in the air pressure and the viscosity coefficient of the air lead to the change in the squeeze-film air damping in the micro-accelerometer based on a silicon carbide microstructure. When the micro-accelerometer vibrates in the rarefied air, damping is the main form of air film in low-frequency vibration, and stiffness is the main form of air film in high-frequency vibration. Both the viscous damping force and the elastic damping force of the air decrease with the decrease in the air pressure. As the micro-accelerometer works at a low vibration frequency, the viscous damping force is the main source of its air damping force.

It was found that with decreases in the air pressure and the viscosity coefficient of the air, the corresponding damping coefficient and relative damping ratio of the micro-accelerometer decreases. A physics-level simulation was proposed in Ansys/Fluent to simulate the squeeze-film effect of rarefied air in the comb structure of the micro-accelerometer. The simulation results agree well with the theoretical analysis and effectively verify the theoretical analysis of the squeeze-film damping in rarefied air.

The changes in the damping coefficient and relative damping ratio of the micro-accelerometer will directly affect the micro-accelerometer’s dynamic characteristics, including amplitude–frequency characteristics and step responses. Therefore, the micro-accelerometer in the rarefied air was analyzed in the frequency and time domains. When the air pressure of the working environment is below the standard atmospheric pressure, the micro-accelerometer will be in an underdamping state. It can be seen from the analysis in the frequency domain that with the decrease in the air pressure, the working bandwidth of the micro-accelerometer will decrease significantly, and the resonant peak will appear in the amplitude–frequency characteristic curve; that is, the resonant phenomenon may appear. It can be seen from the analysis in the time domain that the response time of the micro-accelerometer will increase with the decrease in the air pressure. However, as it is still in the same order of magnitude (10^−4^ s), the decrease in the air pressure will not have a notable impact on the response time of the micro-accelerometer.

## 6. Conclusions

In this work, we investigated the viscosity, the squeeze-film effect, and a SiC-based capacitive accelerometer in rarefied air. When the air pressure drops from the standard atmospheric pressure to 12,400 Pa and 1243 Pa, the effective viscosity coefficient of the air decreases by about 4.1% and 39.9%, respectively. The decreases in the air pressure and the viscosity of the air lead to the decrease in the relative damping ratio (ς=0.66 and ς=0.41) of the micro-accelerometer, which will directly affect the dynamic characteristics (amplitude–frequency characteristics and step responses) of the micro-accelerometer. When the air pressure in the working environment is below the standard atmospheric pressure, the micro-accelerometer will be in an underdamping state. With the decrease in the air pressure, the working bandwidth of the micro-accelerometer will decrease significantly, and the resonant phenomenon may appear. However, the decrease in the air pressure will not have a considerable impact on the response time of the micro-accelerometer. Therefore, this work is of great significance for the study of the performance characteristics of SiC-based capacitive micro-accelerometers in rarefied air.

## Figures and Tables

**Table 1:** The structural parameters for the capacitive micro-accelerometer [21].

Accelerometer	Length (µm)	Width (µm)	Thickness (µm)
Mass block	500	350	20
Movable electrode plates	300	4	20
Fixed electrode plates	300	4	20
Folded support beams	350	4	20

**Table 2:** The changes in the relevant parameters of the air when the air pressure drops.

Air Pressure	101,325 Pa (1 atm)	12,400 Pa	1243 Pa
Mean free path (m)	2.4537 × 10^−8^	2.005 × 10^−7^	2.0001 × 10^−6^
Knudsen number	0.0012	0.01	0.1
Effective viscosity coefficient (N·s/m^2^)	1.7821 × 10^−5^	1.7098 × 10^−5^	1.0716 × 10^−5^
Decline ratio (%)	-	4.1%	39.9%

**Table 3:** Simulation parameter settings under low pressures.

Parameters	12,400 Pa	1243 Pa
Acceleration of gravity (m/s^2^)	9.8	9.8
Air density (kg/m^3^)	0.15	0.015
Effective viscosity coefficient (N·s/m)	1.7098 × 10^−5^	1.0716 × 10^−5^
Environment temperature (°C)	15	15
Speed of the moving plate (µm/s)	10	10

**Table 4:** Comparisons between theoretical values and simulated values of two parameters for the comb structure under low pressures.

Structure	Parameter	12,400 Pa		1243 Pa	
		Theoretical Value	Simulated Value	Theoretical Value	Simulated Value
Comb structure	Air damping force (N)	2.04 × 10^−10^	1.96 × 10^−10^	1.28 × 10^−10^	1.23 × 10^−10^
	Damping coefficient	2.04 × 10^−5^	1.96 × 10^−5^	1.28 × 10^−5^	1.23 × 10^−5^
Accelerometer	Air damping force (N)	8.16 × 10^−9^	7.84 × 10^−9^	5.12 × 10^−9^	4.92 × 10^−9^
	Damping coefficient	8.16 × 10^−4^	7.84 × 10^−4^	5.12 × 10^−4^	4.92 × 10^−4^

## Data Availability

Not applicable.
